# The factitious/malingering continuum and its burden on public health costs: a review and experience in an Italian neurology setting

**DOI:** 10.1007/s10072-021-05422-9

**Published:** 2021-08-04

**Authors:** Marco Onofrj, Anna Digiovanni, Paola Ajdinaj, Mirella Russo, Claudia Carrarini, Massimo Di Giannantonio, Giovanni Martinotti, Stefano L. Sensi

**Affiliations:** 1 Department of Neuroscience, Imaging, and Clinical Sciences, “G. D’Annunzio University” of Chieti-Pescara 66100, Chieti, Italy; 2 Center for Advanced Studies and Technology (CAST) “G. D’, Annunzio University” of Chieti-Pescara 66100, Chieti, Italy; 3 YDA Foundation, Institute of Immune Therapy and Advanced biological treatment, Pescara, Italy; 4 Department of Clinical, Pharmaceutical and Biological Sciences, University of Hertfordshire, Herts, UK

**Keywords:** Factitious disorders, Malingering, Functional neurological symptoms disorder, Hysteria, Munchausen’s syndrome

## Abstract

Factitious disorder is classified as one of the five aspects of somatic symptom disorders. The fundamental element of factitious disorder is deception, i.e., pretending to have a medical or psychiatric disorder, but the enactment of deception is considered unconscious. Indeed, volition, i.e., the perception of deliberate deception, is blurred in patients presenting with factitious disorder. In the USA and the UK, factitious disorder has received constant media attention because of its forensic implications and outrageous costs for the National Health Systems. Unfortunately, a comparable level of attention is not present in Italian National Health System or the Italian mass media. The review analyzes the classifications, disorder mechanisms, costs, and medico-legal implications in the hope of raising awareness on this disturbing issue. Moreover, the review depicts 13 exemplification cases, anonymized and fictionalized by expert writers. Finally, our paper also evaluates the National Health System’s expenditures for each patient, outlandish costs in the range between 50,000 and 1 million euros.

## Definitions and red flags

The factitious disorder is classified by DSM-5 [1] as one of the five aspects of somatic symptom disorders. This is at difference with the DSM IV-TR [2] version, which coded factitious disorder outside of somatoform disorders and edging dissociative disorders. Although the fundamental element of factitious disorder is deception, i.e., pretending to suffer from a medical or psychiatric disorder, the enactment of deception is also considered unconscious. Indeed, the volitional aspect, i.e., perception of deliberate deception, is blurred in patients suffering from factitious disorders. There is no apparent economic purpose in patients with factitious disorder, at difference with malingering, which is considered willed fraudulent behavior [1, 2].

The disorder reached a definite identity only in the DSM III [3]. Therefore, related interpretations are likely biased by the previous insufficient separation from other somatic symptoms disorders and malingering. Before the DSM III, factitious disorder and malingering were considered present mainly in the military (drafted personnel) and criminal world [4, 5]. For some authors, a catalyst of factitious disorder and malingering was the creation of social welfare and the access to financial compensations or unnecessary care [6].

According to the early presentation of the disorder [7], in the DSM IV, factitious disorder was also termed Munchausen syndrome. The DSM-5 now disfavors its use. The reasons to opt for other terms are discussed in detail by Feldman and Yates [6], where attention is focused on the need to highlight the abusive behavior in legal terms.

The core definitions of factitious disorder are that “are conditions in which a patient intentionally produces or feigns physical or psychological symptoms…without obvious secondary gain (ICD-10 definition).” [8] The DSM-5 states that “the motivation for the behavior is to assume the sick role” but, although deception and feigning are the core element for the diagnosis, the manuals also indicate that “assessment of conscious intention is unreliable” and diagnosis is linked to the chance to incur into evidence of feigning [1]. Therefore, guidelines indicate that deception and the absence of external incentives for the behavior are diagnostic criteria. However, paradoxically, the same guidelines state that the intention of feigning cannot be reliably assessed. The DSM IV-TR [2] also provided a detailed guide (red flag, indicating that the presence of specific behaviors should alert the clinics to suspect a factitious origin) for differential diagnosis. “Suspicion that an apparent mental disorder or general medical condition in fact represents factitious disorder should be aroused if any combination of the following is noted in a hospitalized individual: an atypical or dramatic presentation that does not conform to an identifiable general medical condition or mental disorder; pseudologia fantastica (pathological lying); disruptive behavior on the ward (e.g. noncompliance with hospital regulations, arguing excessively with nurses and physicians); extensive knowledge of medical terminology and hospital routines; covert use of substances; evidence of multiple treatment interventions (e.g. repeated surgery); extensive history of traveling; a fluctuating clinical course, with rapid development of “complications” or new “pathology” once the initial workup proves to be negative.”

Factitious disorder may also appear as a “by proxy” behavior, termed as “Munchausen Syndrome by proxy” [9]. In this case, the quest for medical attention is transposed to people (often children) cared for by the person with factitious disorder. Using drugs or physical manipulations or direct poisoning, the perpetrator induces sick conditions to persons of whose care he/she is charged. Recently, a substitution of “Munchausen Syndrome by proxy” with “Medical Child Abuse” has been proposed with the aim, again, to address the legal aspects of the condition [10, 11].

Because pathological lying (pseudologia fantastica) is a critical component of the disorder, it is argued that the clinician should actively seek its identification. Pathological lying is distinguished from “normal” lying by several characteristics, including recurrent, enduring, and compulsive lie presentations as well as fantastic, self-aggrandizing content and the possible ego-dystonic structure with maladaptive or destructive outcomes for the quality of life of the pathological liar [12, 13].

Factitious disorders may appear in association with other mental disorders. The DSM-5 [1] quotes the association with the other four somatic symptoms disorders (i.e., the somatic symptoms disorder-Briquet syndrome, the conversion-functional neurologic disorder, illness anxiety disorder-hypochondria, psychological symptoms occurring during other medical conditions) and with dissociative disorders. The DSM IV-TR [2] includes histrionic, antisocial, borderline, and dependent personality disorders. The DSM-5 goes to the extent of stating that “some aspects of factitious disorders might represent criminal behavior.”

The recent neurologic literature has proposed a clear-cut separation of conversion/functional neurologic disorders (FND) from factitious disorders [14, 15]. However, as mentioned above, the DSM classification system underlines the frequent overlap between different forms of somatic symptom disorders, once termed hysteria and, therefore, a hystero-malingering continuum has been suggested [12, 16].

Studies analyzing the occurrence of somatizations in adopted patients and their biological parents indicated that the somatization disorder was associated with heritable personality traits like predisposition to antisocial behavior and substance abuse [17–19]. Thus, the antisocial trait (and possible further evolutions to criminal behavior) is an aspect of the factitious/malingering continuum that should not be overlooked.

Factitious disorder is not rare; epidemiological studies show that 1% of referrals to psychiatry liaison services in hospitals exhibit the disorder [20]. Around one-third of the perpetrators of medical child abuse (by proxy disorder) have factitious disorder themselves [21].

Factitious disorder has been extensively studied in the USA and the UK. These studies have highlighted the outrageous costs to the healthcare systems [20, 22]. In Italy, less than 40 single case reports have been published so far, all exclusively reporting “by proxy” cases of medical child abuse [23]. (results of PUBMED search for, Factitious, Munchausen, Italy).

The present study aims to call attention to this disorder and estimate the costs to our healthcare system. The cases reported here are taken from our Neurology Clinic, but factitious disorders have a great impact on the clinical practice of all the other medical disciplines. For instance, factitious disorder commonly overlaps with brittle diabetes, *dermatitis factitia*, and gastroenterological presentations [18, 24].

## Psychodynamic mechanisms

Psychodynamic interpretations describe primary gain (i.e., the solution of an intrapsychic conflict) as the origin of factitious disorder. In contrast, secondary gain, which is the practical or economic benefits resulting from the enactment of specific behavior, is typically associated with malingering [25]. Primary gain has been described as keeping an internal conflict or need out of personal insight. Secondary gain is instead associated with avoiding a particular activity that is noxious. Secondary gain also aims at getting support from the environment that otherwise might not be forthcoming. However, the primary gain has also been linked to Conversion disorders, to the point that several studies depict a continuum [10].

Psychodynamic studies highlight that “factitious disorders are famously difficult to treat medically and are highly refractory to psychotherapy.” [25] Only a few reports have successfully described the interaction between the therapist and patient affected by factitious disorder; many authors have concluded that genuine communication is difficult and almost always burdened by deception and opposition. Some authors have also described confrontational management [18] issues once deception is unveiled. Many authors underlined oppositive and defiant responses to attempts to rationalize and explain the behavior to the patient. An unfruitful attempt, constantly resulting in the patient’s question: “what if that’s true?” [8, 20].

An intriguing report, produced from a rare collaborative patient, described herself in a psychotherapy session as “desperate to try and get help” [18]. She wrote: “I despise myself for all the things I have done, and have endlessly tried to stop what it is like an addiction.” Similar descriptions can also be found in a recent book by Feldman and Yates [6].

The unconscious origin of the feigning behavior is interpreted as the hidden drive to be in control of medical conditions that were previously experienced as painful. In this respect, these disorders can also be viewed as repetitive compulsions motivated by the desire to dominate and master (i.e., taking control over) the medical personnel providing care [25]. Feigning, or inflicting damages to a proxy (performed in a state of blurred consciousness), can be interpreted as the expression of unconscious wishes to enact a personal drama and reinforce the strength of a relationship with medical professionals who live in the fantasy of the patient [18, 26]. In the 1978 draft of the DSM III, [3] the underlying motive of factitious disorder was presented as the *compulsion to act out a sadomasochistic relationship with physicians who are regarded as parental figures* [6]*.*

A current revaluation of the majority of psychodynamic interpretations produced before that DSM III [3] often unveils a mixture of somatic symptoms (conversion or functional neurologic disorders (FND)) and factitious disorder exhibited by the same patient (hence the reiterated concept of a continuum) [16, 18, 20].

The psychodynamic model of reference invokes an unconscious mechanism that acts independently of the patient consciousness, thereby favoring the assumption that a disordered unconscious might give rise to a disordered will [25].

From these hypotheses, an unconscious intention to action would rely on a simultaneous (conscious) intention to act. Within this frame, Lacanian interpretations introduced the concept of “failed acts” either as identification with an external will or as “acting in” within a fantasized body space to explain the production of FND motor symptoms or the complex activities enacted in factitious disorder [27].

Further interpretations were focused on self-deception, avowal, and disavowal of action. These suggested that disavowal of action [28] is acted to avoid disturbing the patient’s image of his/herself. Thus, the action becomes not intended as its access to consciousness is barred [28–30]. The concept of avowal could not separate FND from feigning. Unsurprisingly, the dissection of volition led to discussing moral contents, distinguishing an unconscious, morally neutral deception where it is the self and not the other, whom the deceiver is deceiving, from a deception being a willed act, which should then be pure malingering.

**Fig. 1:**
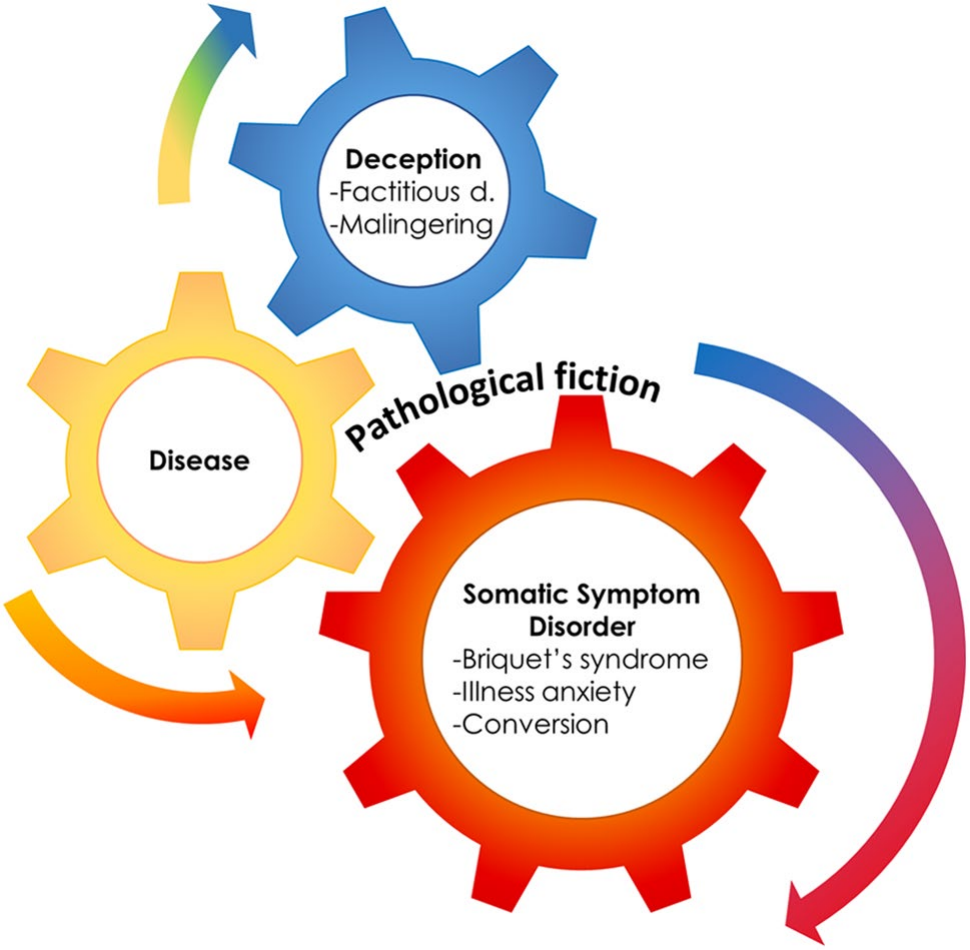
The presence of a medical condition can often mesh with deception and somatization. The fictionalized cases presented in the paper, unveil a mixture of somatic symptoms (conversion or functional neurologic disorders (FND)) and factitious disorder that co-exist in the same patients, thereby supporting the hypothesis of a hystero-malingering continuum. Reproduced, with modifications, from Feldman and Yates, 2018

These interpretations blurred the boundaries between FND and factitious disorder, and, again, a possible continuum was suggested, termed “hystero-malingering continuum” [12] (Fig. 1). For example, it was shown that hysteric patients misinterpret evidence and selectively pay attention to only a specific part of the overall evidence. Patients who employ such strategies as positive and negative misinterpretations, selective attention, and selective evidence gathering are biased (driven by primary, secondary, and economic gains) and can become, therefore, self-deceiving [29].

## Other potential mechanisms

First in philosophy (as in work by Hume) [31], and later in psychology, three main elements of voluntary (willed) actions were considered. These elements are either (a) *volition*, or will, defined as the power to order the forbearing of an idea; (b) *agency*, as the perception of being the one who chooses or enacts the action; and (c) *intention*, as the conscious choice generated by the agent of the action. For example, intention, volition, and agency to act but no motor effects are present in organic paresis. Conversely, in utilization and imitation behaviors (driven by frontal dysfunction), no intention or volition is intended, whereas agency is perceived. In some sensory deafferentation conditions, like the posterior alien hand syndrome, the sense of agency is lost.

Based on these three categories, several attempts have been made to demonstrate structural or functional correlates of feigned acts or motor disorders ascribed to psychogenic causes.

Initially, the focus has been on volition “they say, I cannot; it looks like I will not; but it is I cannot will.” Therefore, manifestations of unconscious actions were interpreted as driven by disordered will (Paget, 1873) [32].

More recently, the focus has shifted on altered agency [15, 33, 34], which was considered the expression of disordered perceptions and altered by insufficient suppression of priors recruited by top-down perception processing [33–35].

Attention to the motor act, as part of intention, has been also invoked, to explain the distractibility of FND and feigned motor disorders. It must be pointed out that when invoking the concept of attention, a paradox surfaces. The maintenance of unconsciously derived FND symptoms requires in fact conscious attention [36, 37]. The main diagnostic method for dissecting out a functional motor disorder is based on testing the distractibility of the symptom, i.e., if the patient is distracted, the functional or feigned symptom must disappear. However, this finding also indicates that attention is needed to produce the symptom. As attention is part of conscious behavior, the paradox emerges. Indeed, contrary to the construct’s supporting assumption, it is the patient insight and awareness that maintain the symptom’s production. The same interpretation can be applied to the agency of the act of feigning in factitious disorder.

Different reports have suggested dysfunctions in top-down processes. However, the lack of statistically powered studies makes these findings still largely anecdotical [14, 15, 33, 38].

More recent interpretations suggested that factitious disorder should be considered based on the mechanisms of addiction or duress (i.e., like in pathological gambling) [39].

In patients with factitious disorders, the agency of feigning and volition of feigning is denied and erased from consciousness; intention should be consequently unconscious.

Much needed research should be invested to identify the neural substrates of deception in factitious disorder, an ambitious target not achieved so far [20, 40, 41].

## Anonymized representative cases

All cases described here went through anonymization and have been fictionalized by expert writers (age, gender, profession, history, initials, are all fictional. The original observational studies were approved by the local ethical committee (number 16 of 19/Jul/2018 ,emendment 7/May/2020 and number 2098 of 11/Jun/2020).

### Case 1: AA male, 35 years old

After four previous admissions in other Italian Neurology Clinics, AA came to our observation, asking for muscle and nerve biopsy. The patient complained of leg weakness, with the impossibility to walk and stand, and unclear sensory disturbance in the absence of pain. He specifically claimed to suffer from an unknown muscle or peripheral nerve disease. Objective neurological examination showed normal segmental strength, normal trophism, and normal deep reflexes. Electromyography/neurography (EMG/ENG) and motor and sensory evoked potentials were normal. The medical history revealed previous litigation for medical malpractice (abdominal surgery) that resulted in monetary compensation at the age of 18.

The patient’s attitude was highly hostile with doctors and nurses. He also attempted manipulatory strategies with the medical staff and repeatedly threatened to start a malpractice suit. One night, he was eventually found by nurses standing in the bathroom.

Immediately dismissed, with a diagnosis of Munchausen syndrome, he still managed to arrange a private transferal to another neurology clinic. In the following year, he was admitted to four more neurology clinics, always pretending to be cured and studied for his unknown disease. Meanwhile, he obtained nationwide attention from several newspapers and calling for an effort of the scientific community to find a diagnosis. After almost another year of repeated hospital admissions, he disappeared from media and social network websites.

In 18 months, AA totalized 370 days of hospital stay and underwent repeated clinical and instrumental examinations, with an estimated cost for the community of 512,000 euro, this notwithstanding the cost of the emotional stress for personnel staff.

### Case 2: BB, female, 54 years old

BB was the mother and caregiver of a 26-year-old girl affected by refractory epilepsy. At the age of 4, the girl had presented with seizures, probable partial or generalized status, which were not recognized by the pediatrician. For this reason, BB had obtained congruous compensation from the hospital. Since then, she toured several epilepsy clinics in the country, asking for different interventions, including epilepsy surgery of different kinds.

When she came to our clinic, the girl had undergone bilateral temporal lobectomy, callosotomy, tracheostomy, and percutaneous gastrostomy (PEG), and was unable to stand unaided. BB’s daughter was under treatment with phenobarbital, clobazam, felbamate, carbamazepine, valproate, and vagal stimulator. BB complained that the girl was still suffering from seizures or status. Ingestion pneumonia was detected at admission. During the hospital stay, BB pretended to be the only one to administer drugs to the girl.

Her attitude was extremely hostile and aggressive. She also expected to have constant attention, tried to foster rivalries among the doctors, and violently targeted some doctors. On repeated occasions, she physically attacked a neurologist, two nurses, and a pneumologist.

During the stay, therapy was adjusted, reduced to felbamate and stiripentol, and seizure frequency decreased to less than once a week. The patient’s level of consciousness improved, with non-verbal interactions, also with the resolution of pneumonia, and a genetic diagnosis of Dravet’s syndrome was provided.

BB was then sued by the attacked doctors and by the head of the clinic. A month after dismissal, we were informed that the girl had died because of pneumonia. We eventually got to know from the welfare assistants that BB was feeding her daughter with solid food per os, despite every warning.

The diagnosis was Munchausen by proxy. There is still uncertainty on which would have been the girl’s conditions if the mother had not succeeded in obtaining the multiple surgical procedures.

The cost of medical procedures is estimated to exceed 1 million euros.

### Case 3: CC, female, 28 years old at first referral, now 52 years old

CC came to our observation because of seizures, which had been classified as “jocular” by the most prominent epileptologist of the time. Never any epileptic activity was documented by Electroencephalogram (EEG). During the first and subsequent stays, she reported being affected by dissociative identity disorders, with three different personalities coexisting in her body at different times. One was a wild, sexy, uncontrolled personality, accustomed to having all her wishes fulfilled. One was a cultivated lady, a professor of literature, and the third was a low social level housewife.

In her first stay, CC jumped through the clinic’s glass door, attacked the nurses and doctors, and presented with 2–5 psychogenic non-epileptic seizures (PNES) per day. After a thorough diagnostic workout, no evidence of seizures was documented. CC slowly began to manifest her inner beliefs, always exhibiting a defiant attitude and constant menaces of suing the doctors for malpractice. Although emergency treatments with haloperidol or chlorpromazine had, temporarily, contained her histrionic behaviors, she refused any chronic drug or psychotherapeutic treatment.

Her disruptive behaviors prompted, throughout the years, several other admissions. CC filed several accusations of malpractice and requests for compensation.

She was followed by the social services of the clinic for more than 30 years, as she had been abandoned by the family and lost any economic support.

The diagnosis was histrionic personality disorder and Munchausen syndrome.

The total cost of her hospital admissions was more than 600,000 euros.

### Case 4: DD, female, 39 years old

After a minor head trauma due to a traffic accident, DD presented with amnesias, confusional state, dreamy states, and fugues, for which she refused to return to work and persuaded a lawyer to sue for dementia secondary to head trauma.

Extensive workouts could not document any brain lesion. At the visits, she was hostile and defiant, menacing legal actions, and pretended to have several neurologic disturbances, including absences, postural instability, knee buckling, and amnesia. During a PNES, she presented with the hysteric arch and pelvic thrusting.

Repeated attempts to obtain medical and legal attention persisted for 18 months, even though she had already returned to her work. She refused medical and psychotherapeutic treatments.

The diagnosis was malingering and factitious disorder.

The cost of her hospital stay, medical workup, and procedures was 50,000 euros.

### Case 5: EE, female, 42 years old

EE reportedly suffered from (undocumented) epilepsy with less than two seizures per year since her childhood. She had been treated with valproate and carbamazepine until the age of 24, without any recurrence. At the age of 41, EE had accepted to follow her husband, who had been offered a career promotion, to an overseas subsidiary of the central factory. In the new country, she started presenting with convulsive and non-convulsive seizures, with increasing frequency (from weekly to several times per day). Interictal EEG showed right frontotemporal sharp waves and isolated spike-slow waves. Therefore, treatment was reintroduced with lamotrigine, followed by topiramate.

At the admission to our clinic, the extensive workout only documented PNES, with no ictal discharges during video-EEG. Magnetic resonance imaging and interictal perfusion, single-photon emission computed tomography results were normal. The seizure’s phenomenology changed progressively with time. DD started to present fugue states with automatisms, accompanied by oppositive behaviors and falls, and rolling on the floor.

The diagnosis of PNES was not accepted by the patient and, initially, also by the husband. She refused psychotherapy.

The cost of ascertainments and hospital stays was 50,000 euro.

The diagnosis was factitious disorder. However, the secondary gain was achieved as she forced her husband to return to the country of origin. The follow-up was, nonetheless, unfortunate, with a divorce occurring after 3 years.

### Case 6: FF, male, 36 years old

FF presented with paraparesis due to a workplace accidental fall. After the lower back trauma, only an L2 internal rim of fracture was documented at MRI. FF then filed a legal action for reimbursement. The suit was opposed by the National Institute providing coverage for inability resulting from trauma at work, but the opposition was countered by several appeals, protracted for 5 years. During this time, paraparesis converted to paraplegia, and urinary incontinence appeared, which prompted catheterization.

At the evaluation in our clinic, FF presented on a wheelchair with leg extensions, a condition which he claimed had lasted for 5 years. The neurological examination showed normal segmental strength, absent plantar reflexes, and ankle clonus (three jerks) only on the right. EMG/ENG, MEP, and SEP were normal. Trophism examination only showed mild redness of the skin over the sacrum, no decubitus. Urine samples were completely normal, despite the pretended use of permanent catheterization.

The diagnosis was malingering-factitious disorder. However, FF’s lawyers obtained a settlement with the insurance company to end the repeated appeals. The reimbursement was for 300,000 euros, 70% of which went to the lawyers.

### Case 7: GG male, 46 years old

GG presented with right eye optic neuritis (ON) at the age of 40. The acute phase was timely treated with boluses of methylprednisolone and subsided over 15 days. The recovery at 6 months seemed incomplete, with the persistence of a superior altitudinal field defect. However, this picture was more consistent with sector ischemic optic neuropathy than with inflammatory ON. Repeated MRI brain scans only showed persistent hyperintensity of the inferior right optic nerve at STIR, LTIR, and FLAIR sequences. No demyelinating lesions were ever observed within the brain or the spinal cord.

During the following 5 years, GG presented with 12 more episodes of right or bilateral orbital pain, frontal pain, neck pain accompanied by severe agitation, stress reactions, and hostile attitudes. Moreover, also his wife aggressively addressed the doctors, pretending to have immediate treatments and attention for her husband, menacing to use her acquaintances in various national newspapers to divulge on the press the “negligent” treatment.

Repeated visual field examinations showed inconsistent and variable/remitting changes, and visual evoked potentials (VEP) did not show any change after the first episode. Moreover, event-related potentials showed a P300 potential elicited by rare stimuli, also when he was pretending to be blind from the right eye. Screening for anti-aquaporin and anti-MOG antibodies, along with genetic studies for Leber’s hereditary optic neuropathy, was negative.

Nevertheless, GG’s pain episodes and his wife’s menaces continued. He finally succeeded in getting a written diagnosis supporting severe visual impairment and used it to obtain economic support and pension.

The diagnosis was Munchausen by proxy associated with chronic paroxysmal hemicrania, and panic attacks, in prior ischemic optic neuritis. Psychiatric treatment was refused.

The costs of repeated access to the multiple sclerosis unit and unnecessary methylprednisolone boluses (until the diagnosis was ascertained and treatment denied) were 25,000 euros. The cost of a pension for severe blindness, if approved, would be 12,000 euros per year, to be extended for his life expectancy.

### Case 8: FF, female, 58 years old

At the age of 50, FF presented with distal leg weakness and lower limb areflexia. After a complete workout, she was then diagnosed with chronic inflammatory demyelinating polyneuropathy (CIDP) and treated with methylprednisone followed by intravenous immunoglobulin (IVIG) infusions.

After the initial clinical improvement, lasting for 6 months, FF complained of the disorder’s relapses and worsening of the distal weakness. The conduction parameters at ENG were not changed (a possible outcome, as described by literature, which could be disjunct from clinical improvement). The amplitude of compound muscle action potential was not furtherly decreased. However, because of the subjective, untestable complaint of a relapse, a new IVIG course was administered, followed by clinical improvement. From the second treatment, FF complained of further relapses with a yearly/biannual frequency and received treatments (despite some clinicians’ opposition, of which the patient was not informed).

In the following admissions, she became the terror of the clinical unit. She exhibited continuous complaints about delays in treatment, inadequate attention from doctors and nurses, inadequate room furniture quality, poor entertainment during the time spent in the treatment unit, and excessive noise. She successfully filed several written complaints to the hospital administration, to the press, to the “Tribunal of Patients’ Rights”. She explained that her complaints were made to improve the service for everybody and often harangued the other patients waiting for treatment, inviting them to produce a “class action.” She refused any psychiatric approach and treatment.

Her hostile and viscous attitude prompted a diagnosis of Munchausen syndrome in narcissistic personality disorder.

The cost of IVIG treatments was 240,000 euros, and the emotional cost of his continuous complaints and harassment was incalculable.

### Case 9: GG, male, 69 years old

Affected by bipolar disorder, once condemned for sexual harassment, GG was in treatment with lithium and haloperidol. He was admitted to our Neurology Clinic for iatrogenic Parkinsonism, which rapidly subsided with therapy adjustment. Three years later, he had a traffic accident with head trauma, but a neurologic evaluation performed 6 months later showed no pathologic signs.

However, his daughter, a lawyer, filed a compensation request for dementia and Parkinsonism due to the head trauma. Subsequent visits showed severe bilateral Parkinsonism, with tremor, bradykinesia, and postural instability. The evaluation also documented bradyphrenia, untestable mental status, and cognitive levels due to lack of collaboration, dysarthria, poor word articulation, and hyperphonia. The discrepancy of the dysarthria type with Parkinsonism and his medical history prompted in-depth neuroimaging studies. These showed medial temporal atrophy of grade 2 and reduced binding in the left striatum at Ioflupane 123I DAT-scan, which were deemed irrelevant for the litigation.

At the visit, the neurologist suspected that dysarthria and bradyphrenia were due to concealed administration of neuroleptics. The daughter, who was also in charge of medications, denied any administration of neuroleptics and threatened all kinds of legal actions against the clinical and forensic doctors.

Despite a clear report by the neurologist, the daughter succeeded in molesting and menacing the insurance company doctors and lawyers and finally obtained compensation for 500,000 euro.

This case is a mixture of fraudulent behavior and factitious disorder by proxy, deliberately orchestrated to obtain undeserved monetary benefits.

### Case 10: HH, female, 69 years old

HH had been evaluated and treated for hypomania/maniac state and depression episodes 10 to 15 years before the occurrence of a traffic accident, which caused a leg fracture and head trauma with transitory loss of consciousness, no bleeding.

Three months after the trauma, while treated in rehabilitation, she presented with bizarre agitation behaviors, restlessness, aggressivity, and confusional states. She filed a request for compensation for dementia due to head trauma. Once at home, she presented with disinhibited behavior, aggressivity, and fugues. Her behavior prompted several neurologic examinations, which provided contradictory results, with inconsistencies and lack of collaboration during neuropsychological tests. Fluctuating cognition was evidenced, intelligence quotient (IQ) scores varied from 70 to 98, mini-mental state examination score ranged from 10 to 23, and frontal assessment battery scores from 3 to 12. Approximate answers (Vorbereiden) and somatic conversion features (waning left hemiparesis) were also noted, and a diagnosis of Ganser syndrome was provided. HH refused any psychiatric approach, pretending to have the neurologic report canceled.

The litigation with the insurance company ran for 3 years. In the end, she obtained compensation of 200,000 euros and a pension from the National Institute for Accidents at Work due to “frontal lobe lesions.” Four years after the compensation, all the disorders had disappeared.

The diagnosis was for factitious disorder with mimicked Ganser syndrome. The recovery after time suggests that the syndrome was simulated as the Ganser pseudodementia is followed by dementia.

### Case 11: JJ, male, 34 years old at first referral, now 58 years old

At the age of 34, working as a carpenter, JJ fell from a 3-m-high scaffold on his feet. D8 vertebral body fracture was evidenced by computed tomography (CT) scan. He subsequently developed intense pain, urinary retention, and flaccid paraparesis, and was rehabilitated for 1 year. Afterward, JJ recovered from flaccid paraparesis, regained the ability to walk unaided, normal bladder functions, and normal sexual life. However, he complained of severe back pain with myoclonic spasms.

He received monetary compensation for the accident and a temporary pension. He never returned to work, as he complained of continuous, unbearable back pain. For the pain, he was treated by pain medicine units with analgesics, opiates, and finally with a spinal electric stimulator implant. Six years after the implant, he requested to remove the electrodes as he felt that there was no effect and wanted to explore new treatment options. He was then addressed for the first time to our neurology unit. The patient presented in a wheelchair, reporting to be unable to walk for more than two steps. The neurologist found normal segmental strength, normal plantar reflexes, normal tendon, and superficial reflexes. Epicritic and thermal sensitivity was normal, but he complained of severe pain for pinprick stimuli of the back, from level C5 to sacral metamers. Pinprick stimuli and bending of the back elicited diffuse spasms with leg retraction, flexion spasms of the trunk and legs, and *pleurotonus*, lasting for 10 to 40 s. Because of the incongruence between the stimulated metamers and the elicited spasms, the condition was diagnosed as propriospinal myoclonus. The definite confirmation of the functional nature of symptoms came from assessing agonist and antagonist muscle co-contractions at the EMG. It was also supported by the presence of distractibility with entertainment maneuvers and the presence of readiness potentials that preceded the spasms. A revaluation of the personal history and hospital charts confirmed that propriospinal myoclonus had been the only symptom ever presented.

Attempts to explain to JJ the origin of the condition and provide psychotherapy were accompanied by combative opposition and aggression. The patient refused any psychotherapy and physiotherapy. Crossover controls showed that, throughout the years, the patient had kept his driving license, maintained an active social life, and worked, illegally but regularly, in a company owned by a close relative.

In total, compensation for the accident, pain treatments, and disability pension summed up to 1 million euros.

The case represents a mixture of functional and factitious disorder and plain malingering.

### Case 12: KK, male, 46 years old

In his late adolescence, KK presented with sporadic migraine episodes, which increased in frequency to became bimonthly from age 35 to 45. After two distressing life events in his affective and professional life, the headache episodes became daily. The symptom consisted of bitemporal pain, irradiated to the jaw, accompanied by facial spasms and hypophonic voice. Repeated laryngoscopies did not show any abnormality. Facial spasms consisted of normal voluntary activity. Logopedic treatments were unsuccessful. He refused psychotherapy and presented with variegate side effects whenever pharmacologic treatment was attempted. For his disorder, he toured several neurology clinics all over the country, angrily complaining about the inefficacy of treatments. After the last negative laryngoscopy and neck imaging study, he requested a laryngeal muscle EMG, which the neurologist denied. The denial was followed by a worsening of facial spasms and hypophonia, which was reduced to constant whispering. Legal actions aimed at forcing the neurologist to perform the exam and at obtaining compensation for the worsening of symptoms were initiated.

In this case, the initial symptoms were functional and somatic symptoms disorders, but eventually, a factitious disorder emerged. The approximate cost of all the performed exams and visits was about 50,000 euros.

### Case 13: LL, female, 48 years old

LL accessed the neurology clinic after 2 years of repeated neurological evaluations. She lamented weakness (and exhibited dystonic posture) of the left arm and cognitive decline. She, however, normally moved the arm during distracting conversations, and no signs of neurologic disorders were ascertained. CT, MRI, and positron emission tomography (PET) brain scans, as well as lumbar puncture and further proteomics for the investigation of inflammatory disorders, were normal. While on the medical ward, she enacted distressing behaviors, calling for doctors’ and nurses’ continuous attention, threatening to sue for negligent or incompetent medical assistance, complaining of insufficient facilities (Wi-Fi, TV programs, lack of a private room). LL lamented several falls, which were never observed by nurses and physicians. LL also pretended to have delivered medical documents, which were never provided. A search for previous access to medical facilities showed that she had been seen in different hospitals’ medical wards. It also unveiled a complex history that had produced widespread support by social support services. She was the single mother of a 10-year-old girl and a 27-year-old boy who had moved to another town and had not kept contact for 10 years. The 10-year-old girl was compelled to behave like a caregiver, watching for her care, and instructed to call the emergency services at night if she appeared to have respiratory difficulties. Actually, the girl had often called the medical ward, yet never any disorder was observed.

Costs for medical ascertainments and repeated accesses to medical facilities were above 50,000 euro.

This case presented with elements of functional and factitious disorders and borderline personality disorder, and shows the harbinger of a possible “by proxy” disorder.

## Management

General agreement concurs that these disorders are mostly untreatable [25], or that psychotherapy is ineffective, other than being mostly refused. This is not unexpected, given the fraudulent nature of some of the enacted behaviors [25].

However, there is a management issue, as patients who are admitted to medical facilities must be confronted with the diagnosis of factitious disorder. Until now, no better approach was devised than the supportive confrontation of the patient by two or more members of the medical staff, and possibly the involvement of a psychiatric colleague, after careful preparation [6, 18, 42]. Anger, hostility, and irritation, which are common among the clinicians involved, as induced by the hostile attitudes of patients, should be tempered by the preparatory meetings [6, 12].

Face saving is considered the key element for the management confrontation, i.e., it is essential for the patient to be able to explain a recovery, or dismission from a medical facility, to themselves and family members, without admitting the psychiatric nature of the problem [6, 12, 43].

When confronted with the evidence of their deception, patients typically react with denial, aggression, or threats of legal actions. The doctor-patient relationship is irreparable [6].

The concept of *tertiary gain* is also relevant in this context [20, 44, 45]. Tertiary gain occurs when a third subject gains from the perpetuation of the patient’s symptoms. Typical examples include family members who hope to gain financially, physicians who want to recruit patients, and some plaintiff lawyers. Therefore, the confrontation must consider that different sorts of secondary and tertiary gain may be at stake. Even in patients who develop factitious disorder as a defense strategy to get primary gain, a secondary gain may appear as they realize that the illness can bring it. A tertiary gain may follow as part of the surrounding context.

Collecting the evidence for deceit is most often difficult. In the USA and the UK, hidden video recordings (covert video recording, CVR) of patients in their daily life are a common practice and supported by regulatory procedures. In our country, we are unaware of any authorization for video recordings, not even in cases acted with suspected criminal purposes. However, even in the USA and the UK, the attorneys for the accused hospitals or physicians often avoid CVR as they feel that settlements might cost less than a trial, and settlements are frequently reached [46–48].

In the USA and the UK, collateral tests have been developed to reach evidence of deception. These tests are termed “symptom validity tests” or “effort tests” and focused on the ascertainment of pathological lying. In legal parlance, the evidence of lying calls into question the patient’s credibility [20]. One of the most employed is the “coin-in-the-hand test” for patients with amnesia [49]. All are based on probabilistic natures, assuming that any result in a test, which is inferior to results expected by random answering, indicates that the individual intentionally chooses to get answers wrong. These tests, however, may only show the likelihood of willful non-cooperation but cannot prove it [49]. Although these tests have been used, in the USA and the UK, in forensic medicine, as surrogate evidence of lying, their applicability is far from definite.

## The cost on public health and legal systems

The 13 cases provide examples of abnormal behaviors ranging from criminal and fraudulent conduct to unconscious presentations, possibly associated with a grandiose interpretation of oneself and the desire for mastery of medical figures.

The cases also recapitulate the various clinical presentation found in the literature, like “by proxy” behaviors, psychiatric comorbidity, the relevance of previous experiences of compensation, the switch from initial somatic symptom disorder to factitious disorder with belligerent opposition once confrontation was attempted, the overlap/overlay of factitious disorder to coexisting medical (neurologic) diseases, and the blurred borders between factitious and malingering. Of note, it must be considered that unnecessary treatments and investigations may place patients at risk of iatrogenic complications and even death. In American medical literature, cases are reported of patients repeatedly presenting with pseudo-epileptic seizures or status who died because of treatment, as described in a book recently published by the most recognized psychiatric expert of factitious disorder and malingering [6]. For the many cases reported, close similarities with the cases here described are outstanding. The book also provides evidence of cyber-deception [39] or “Munchausen by Internet,” documenting the fraudulent enactment of secondary and tertiary gain, collecting undeserved donations.

In our case descriptions, what was not reported is the amount of time devoted by medical doctors to unnecessary care. It should be a reminder that, in Italy, the National Healthcare system is based on a coding system that categorizes all the diseases divided for each medical specialty (DRG, diagnosis related group) [50]. The coding is key to regulate the amount that the Ministry of Health reimburses to the hospital for any given disease. The DRG does not recognize factitious disorder (nor any somatic symptom disorder) with the neurology category. Therefore, any care provided to patients with factitious disorder is not reimbursed, resulting in a net economic loss. Finally, what is overlooked is the amount of time wasted by medical doctors to defend themselves from illogical or fraudulent allegations, a hidden economic cost neglected by legal litigations.

Our report documents the costs of factitious disorders, which are not adequately recognized by the legal system. It also discusses the immaterial costs driven by inadequate recognition of the disorder or supporting feigned complaints. The inadequate appreciation of the unethical background of factitious disorder forces many medical doctors to shy away from documenting and reporting cases, fearing being left alone and defenseless against aggressive legal prosecutions.

The current cultural climate of the legal system is largely outbalanced in favor of the “victim” role play. Furthermore, threats of legal actions are not discouraged and not prosecuted as the reasoning goes that they cause no harm unless legal action is actually pursued. This interpretation does not take into account the disruption of medical activities that these behaviors cause. This disruption has direct costs, encompassing unjustified procedures, indirect costs for the emotional burden, and the waste of time devoted to providing a shield against fraudulent behaviors.

A revision of attitudes toward factitious disorder must rely on the unethical and anti-economic outcomes of this disrupted behavior, that is to gain undeserved benefits, no matter which the motivation is.

Undoubtedly, as shown by psychodynamic studies, in factitious disorder, borders between volition and insight are dubious and blurred [1, 25]. Still, focusing the attention on practical outcomes rather than dynamics [1] leads to the notion that any complacency with the disordered behavior results in the provision of undeserved gain and outrageous costs and waste of public services and money.

The unethical pretense for an undeserved gain enacted by patients with factitious disorders should be adequately recognized as part of antisocial behavior.

When public health systems’ sustainability is on the brink of collapse, it seems fitting to ask for a shift of the cultural climate of legal systems toward an increased engagement of the medical professions and a proper consideration of immediate and long-term costs of these disorders.

In the USA, it was calculated that the prevalence of factitious disorder/malingering is 10 to 30% of patients seeking compensation, with an estimated cost of $20 billion [20] per year.

As pointed out in the book by Feldman and Yates [6], the legal principles involved in disclosing factitious disorder are equivocal. The Hippocratic principles make no explicit references to medical deception, despite the “How to detect those who feign diseases” was written by Galen of Pergamon almost 1900 years ago [51]. The issue revolves around the authorization to breach patients’ confidentiality in cases of factitious illness. The authors of the book advise seeking legal consultation when danger is predictable and hope for a statement by medical associations, like “In terms of our code of ethics, there are certain circumstances in which confidentiality no longer holds. These include situations in which patients or their caregivers have been fraudulent in producing information and/or have produced the diseases for which they seek treatment.” [6] Legislators must need to develop similar statutory language and judges to affirm its legality. Historically, however, every medical or legal issue has taken priority over consideration of factitious disorders [52].

As a concluding remark, we want to point out that the legal implications of factitious disorder may reach unforeseeable dimensions. Recently, patients with factitious disorder have successfully produced allegations against physicians claiming that by not providing a definite diagnosis, they were responsible for unnecessary medical procedures.

We encourage a shift in attitude and a better understanding of the disorder and its outrageous financial and emotional costs. We also urge raising legal figures  [53] to protect doctors against fraudulent behaviors clearly intertwined with compensation aims as well as against futile allegations presented by plaintiff lawyers.

As a final note, we like to stress that the cases originating our fictionalization were not prosecuted.
